# Intravenous thrombolysis following dabigatran reversal in a patient with acute embolic stroke after atrial fibrillation ablation

**DOI:** 10.1002/joa3.13121

**Published:** 2024-07-22

**Authors:** Yi‐Sheng Chen, Chin‐Yu Lin

**Affiliations:** ^1^ Division of Cardiology Cardiovascular Medical Center, Far Eastern Memorial Hospital New Taipei City Taiwan; ^2^ Division of Cardiology, Department of Medicine Heart Rhythm Center, Taipei Veterans General Hospital Taipei Taiwan; ^3^ Department of Medicine National Yang Ming Chiao Tung University, School of Medicine Taipei Taiwan

**Keywords:** atrial fibrillation, atrial flutter, dabigatran, intravenous thrombolysis, radiofrequency ablation

## Abstract

This case demonstrated the feasibility of administering emergent intravenous thrombolysis followed by Dabigatran reversal with idarucizumab in a patient who underwent atrial fibrillation ablation. The consideration of transitioning anticoagulant therapy to dabigatran for scheduled AF ablation in patients at high risk of stroke should be carefully evaluated.

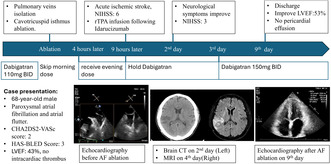

A 68‐year‐old male presented with intermittent palpitations and shortness of breath for 6 months. He has past medical history of paroxysmal atrial fibrillation (AF) with CHA2DS2‐VASc score of 2 and treated with dabigatran 110 milligram twice daily and amiodarone 200 milligram twice daily. Incessant atrial flutter (AFL) and AF was noted for 6 months. Cardioversion restored sinus rhythm 2 months before this admission, but the tachycardia recurred in 1 day. His vital signs were stable, with a heart rate of 120–150 beats per minute. Physical examination revealed an irregular heartbeat, clear breath sounds, and no limb edema. The laboratory data and chest radiograph were normal except for mild anemia (Table 1). AFL was diagnosed by surface electrocardiogram (ECG) (Figure 1A). Transthoracic and transesophageal echocardiography showed the left ventricular ejection fraction of 43%, with no intracardiac thrombus noted (Figure 1B,C).

**TABLE 1:** Laboratory data at admission.

		Units	Reference
WBC	6470	μL	
HGB	10.9	g/dL	
Platelet	157,000	μL	
PT	11.4	s	
aPTT	27.8	s	
INR	1.06		
Creatinine	1.12	mg/dL	0.7–1.2
Na	138	mmol/L	136–145
K	4.5	mmol/L	3.5–5.1
ALT	37	U/L	<41
NT‐proBNP	48	pg/mL	<125

Abbreviations: ALT, alanine aminotransferase; aPTT, activated partial thromboplastin time; INR, international normalized ratio; PT, prothrombin time.

**FIGURE 1:**
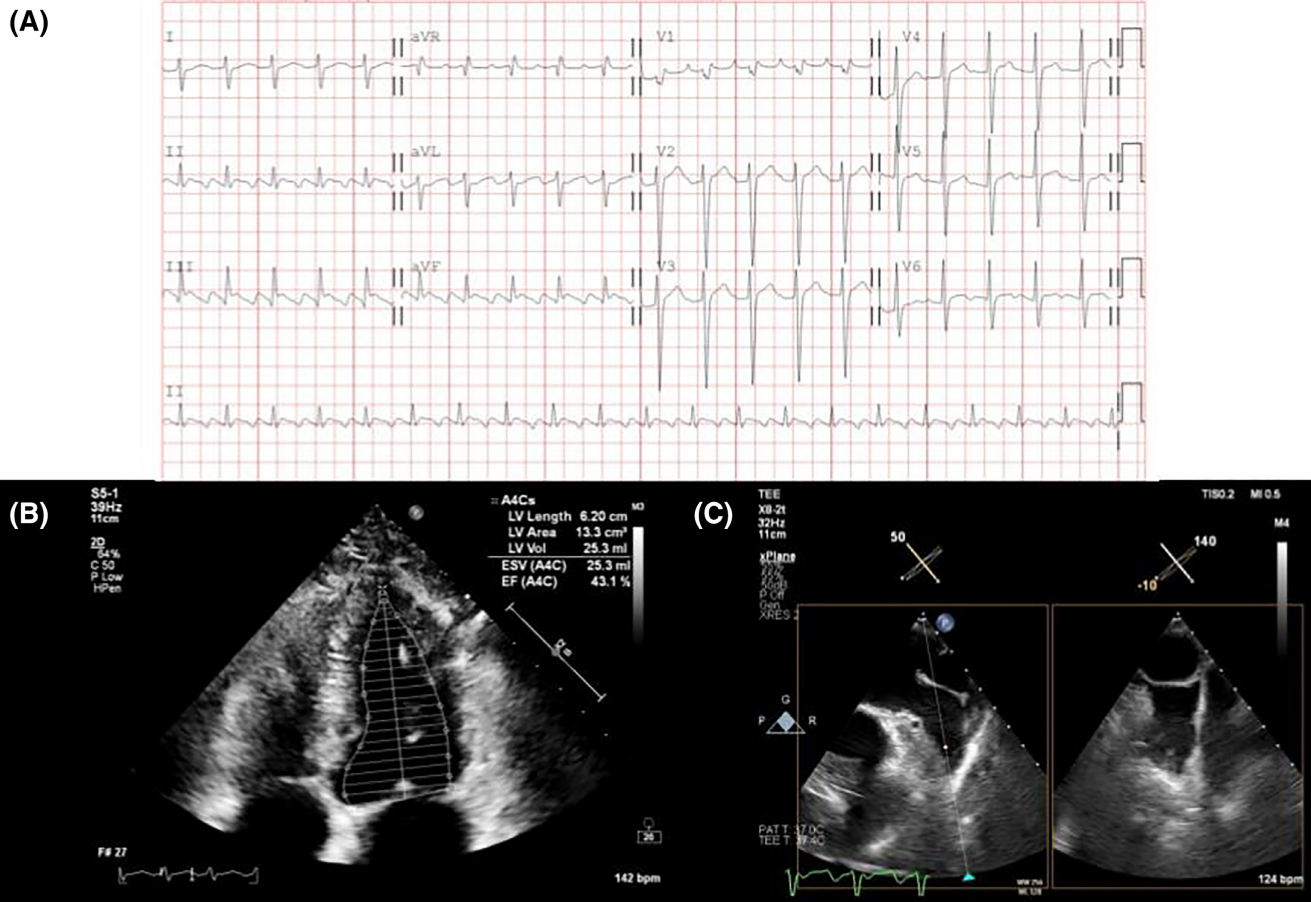
ECG and echocardiography before AF ablation. (A) The ECG showed typical atrial flutter with 2 to 1 ventricular response. (B) Transthoracic echocardiography revealed impaired left ventricular systolic function without regional wall motion abnormality. (C) Transesophageal echocardiography showed no intracardiac thrombus in the left atrial appendage. AF, atrial fibrillation; ECG, electrocardiography.

The patient underwent electrophysiology study under general anesthesia and skipped one dose of preprocedural dabigatran. Intravenous bolus of unfractionated heparin (100 units/kg) was given at the beginning of the procedure and then supplemented according to activated coagulation time (ACT). The ACT was checked every 15 min using a commercial cassette and maintained over 350 s throughout the procedure. Patient's initial rhythm was cavotricuspid isthmus (CTI)‐dependent AFL and terminated after CTI ablation. Subsequently, transseptal puncture was performed, followed by circumferential four pulmonary veins isolation with irrigating ablation catheter under energy settings of 45 W with lesion site index 5.0 and 4.5 over anterior and posterior left atrial wall, respectively. Bidirectional block and pulmonary vein isolation were confirmed, and the inducibility test with isoproterenol did not provoke AF or flutter. The total number of left atrial ablation sites was 109, and the left atrial indwelling time was 74 min. Without heparin reversal, dabigatran was administered 4 h after procedure. Approximately 9 h after the procedure, the patient developed left facial palsy, slurred speech, and left‐sided weakness. A subsequent emergent computer tomography (CT) scan showed no obvious hypodense lesion but revealed scattered defects over the right frontoparietal lobe, lateral temporal lobe, and left frontal and occipital lobes based on the hypoperfusion index ratio (Figure 2A,B). The neurologist was consulted and diagnosed an acute embolic stroke in the right middle cerebral artery with a National Institute of Health Stroke Scale (NIHSS) score of 6, and suggested intravenous thrombolysis after dabigatran reversal. After 6 min following idarucizumab infusion, the patient received intravenous thrombolysis with a standard dose (0.9 mg/kg) of recombinant tissue plasminogen activator (rtPA). On the next day, the patient demonstrated significant improvement, with full recovery of muscle power and a decrease in NIHSS score to 3. A brain CT revealed no signs of intracranial hemorrhage (Figure 2C). 4 days after the AF ablation, magnetic resonance imaging (MRI) revealed scattered infarcts in the right frontoparietal lobe, lateral temporal lobe, temporal operculum, and insula, consistent with an embolic stroke (Figure 2D). The patients restarted dabigatran 150 mg twice daily on the third day after rtPA. He was discharged smoothly on the ninth days after rtPA without sequelae. The ECG showed normal sinus rhythm, and the transthoracic echocardiography revealed an improved left ejection fraction and no pericardial effusion (Figure 3).

**FIGURE 2:**
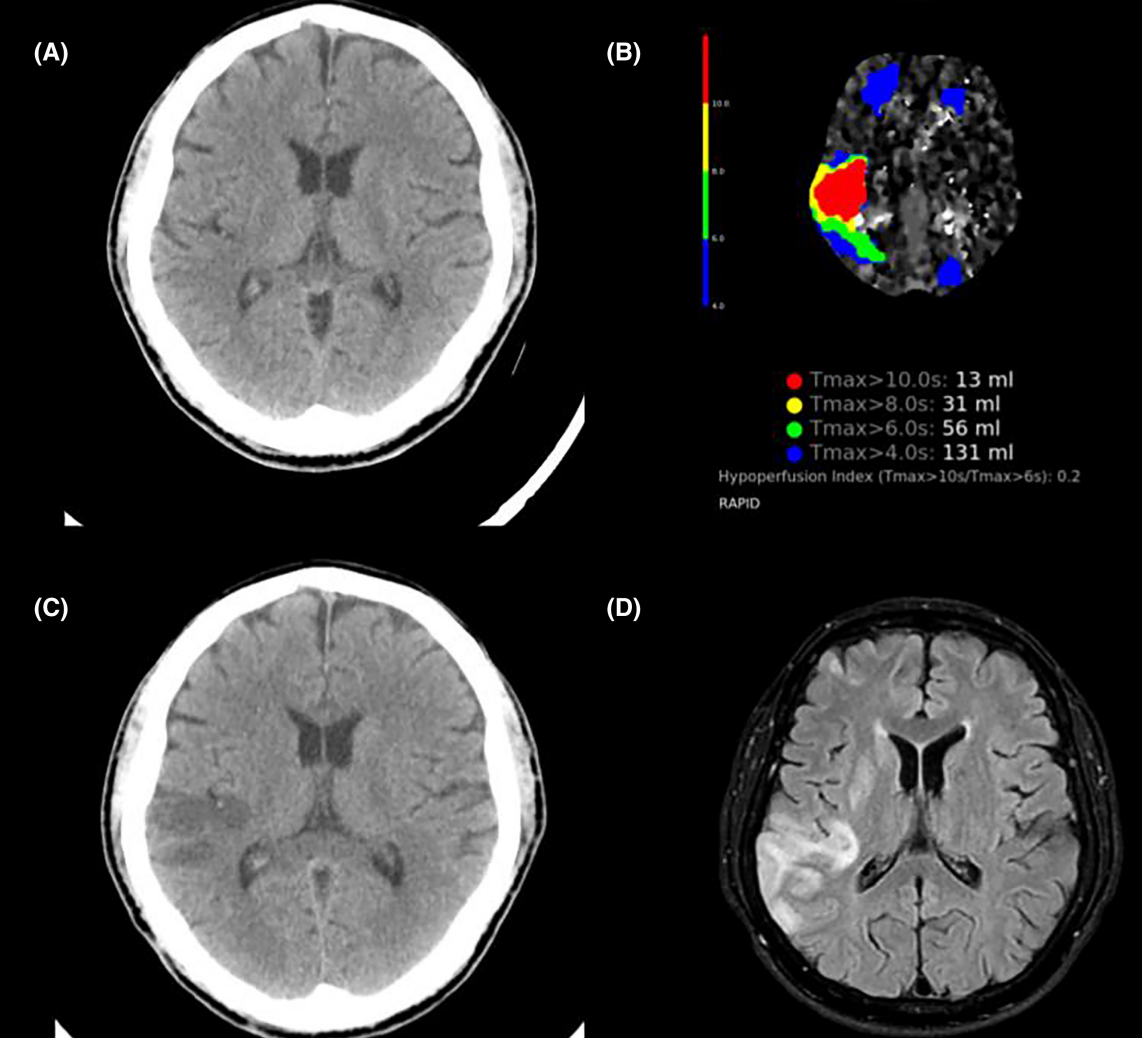
Serial brain images of the patient. (A) The brain CT showed mild swelling in the right lateral temporal lobe without an obvious hypodense lesion. (B) The hypoperfusion index ratio revealed scattered defects over the right frontoparietal lobe, lateral temporal lobe, and left frontal and occipital lobes. (C) The brain CT on the next day following intravenous thrombolysis showed a hypodense lesion in the right lateral temporal lobe without hemorrhagic transformation. (D) The brain MRI with diffusion‐weighted imaging (DWI) revealed scattered infarcts in the right frontoparietal lobe, lateral temporal lobe, and insula. CT, computed tomography; MRI, magnetic resonance imaging.

**FIGURE 3:**
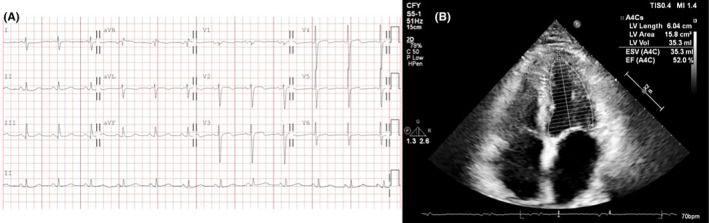
ECG and echocardiography after AF ablation. (A) The ECG showed normal sinus rhythm. (B) The transthoracic echocardiography revealed improved left ventricular systolic function, with an ejection fraction of 53% by the Simpson method, and no pericardial effusion. AF, atrial fibrillation; ECG, electrocardiography.

It is recommended to perform AF ablation without NOACs interruption, as having low incidence of periprocedural thromboembolic events (0.15%–0.5%).^1^ In most postablation cases experiencing periprocedural stroke with an NIHSS score above 6, endovascular thrombectomy is recommended as the first‐line treatment for revascularization. In contrast, intravenous thrombolysis is recommended for NIHSS scores ranging from 4 to 25 and serves as a complementary treatment to thrombectomy for NIHSS scores of 4–6 in postablation patients.^2^, ^3^ However, intravenous thrombolysis is contraindicated in patients who have taken NOACs within 48 h.^2^, ^3^ Dabigatran is the only NOAC with a specific reversal agent, idarucizumab, which is reported having high efficacy of perioperative hemostasis of 98.8%.^4^ Switching NOACs to dabigatran before AF ablation should be considered for high thromboembolic risk patients to facilitate potential periprocedural stroke treatment.

Periprocedural complications of AF ablation peak within 24 h and the risk extends for 2 weeks after procedure, with vascular complications as the common complication following cardiac tamponade and thromboembolic events.^1^ There is limited literature discussing the safety of intravenous thrombolysis following AF ablation. In most cases, vascular complications are easy to exclude before intravenous thrombolysis. However, slow progression of pericardial effusion is difficult to detect, and delayed cardiac tamponade has been reported to develop at a median of 12 days.^5^ The prevalence is estimated to be higher in AF ablation compared with other types of arrhythmias, due to factors such as uninterrupted NOACs, trans‐septal puncture, and extensive manipulation of the catheter.^1^ Therefore, patients should undergo risk stratification and receive intracardiac imaging before and after intravenous thrombolysis.

In conclusion, this case highlights the feasibility of administering emergent intravenous thrombolysis followed by dabigatran reversal with idarucizumab in a patient who underwent AF ablation. The consideration of transitioning anticoagulant therapy to dabigatran for scheduled AF ablation in patients at high risk of stroke should be carefully evaluated.

## FUNDING INFORMATION

This work was supported by the Biosense Webster IIS (C2304900), Ministry of Science and Technology (MOST 110‐2314‐B‐A49A‐541‐MY3, MOST 111‐2314‐B‐075‐007‐MY3), and Taipei Veterans General Hospital (grant no. C19‐027). The funders did not involve the design of this study. C‐YL was the recipient of the funding award. The funders had no role in the study design, data collection and analysis, decision to publish, or manuscript preparation.

## CONFLICT OF INTEREST STATEMENT

All authors have no conflict of interest to declare.

## ETHICS APPROVAL

N/A.

## PATIENT CONSENT STATEMENT

Informed consent was obtained from the patient.
